# Elimination of LMP1-expressing cells from a monolayer of gastric cancer AGS cells

**DOI:** 10.18632/oncotarget.16996

**Published:** 2017-04-10

**Authors:** Yoshitaka Sato, Shingo Ochiai, Takayuki Murata, Teru Kanda, Fumi Goshima, Hiroshi Kimura

**Affiliations:** ^1^ Department of Virology, Nagoya University Graduate School of Medicine, Showa-Ku, Nagoya 466-8550, Japan; ^2^ Division of Microbiology, Faculty of Medicine, Tohoku Medical and Pharmaceutical University, Aoba-Ku, Sendai 981-8558, Japan

**Keywords:** EBV, LMP1, cell competition, cell-cell communication, gastric cancer

## Abstract

Epstein-Barr virus (EBV) latently infects malignant epithelial cells in approximately 10% of all gastric cancers. Latent membrane protein 1 (LMP1), an oncogenic protein, plays an important role in malignant transformation in EBV-associated nasopharyngeal carcinoma and B-cell lymphoma; however, its expression has not been detected in EBV-associated gastric cancer. To address why LMP1 has not been detected in EBV-positive gastric tumors, we focused on the interactions between LMP1-positive and -negative cells and stably expressed LMP1 in the gastric cancer cell line AGS. We showed that the number of LMP1-positive cells decreased gradually with each cell passage when the cells were co-cultured with LMP1-negative cells. Time-lapse imaging showed that LMP1-positive cells were eliminated from a monolayer of LMP1-negative cells. Furthermore, LMP1-positive cells stimulated the proliferation of surrounding LMP1-negative cells, but not LMP1-positive cells, via exosome-mediated EGFR activation. Our data indicate that LMP1 expression drives cell competition between LMP1-positive and -negative cells, affecting the behavior of the cells within gastric tissue.

## INTRODUCTION

Multicellular organisms have evolved tissue homeostasis mechanisms to ensure the fitness of their organs. The mechanisms that regulate the elimination of unwanted cells are fundamental for tissue development and homeostasis [1]. In fact, it has become clear that certain types of transformed cells can be extruded from monolayers of epithelial cells. For example, Madin-Darby Canine Kidney (MDCK) cells expressing oncogenic Ras or Src were eliminated from a cultured epithelial monolayer when surrounded by normal MDCK cells [2, 3]. During the initial stages of neoplasm development, transformed cells emerge as clones surrounded by normal cells, suggesting that normal and transformed cells compete with each other for survival, and intrinsic tumor-suppressive mechanisms depend largely on cell-cell communication to eliminate oncogenic cells [4]. Similar to oncogenesis, infected cells grow in the presence of neighboring normal cells during the initial stage of viral infection. The interactions between infected and surrounding uninfected cells are less understood.

Epstein-Barr virus (EBV) is a herpesvirus associated with B-cell and epithelial cell malignancy. The majority of infected adults are asymptomatic; however, a few develop cancer, with increased risk upon immunosuppression [5, 6]. EBV persists latently in B-cells with sporadic reactivation, and it also infects epithelial cells in the nasopharyngeal area, either directly or indirectly, via EBV-infected B cells [7–9]. Although entry of EBV into the gastric epithelium is still in question [10], malignant epithelial cells are infected with EBV in nearly 10% of gastric cancers [11]. The primary outcome of epithelial infection is lytic infection. Persistent latent infection is associated with the oncogenic phenotype of gastric carcinoma [12]. The pattern of viral latent gene expression (latency I, II or III) is dependent on the tissue of origin and the cell state [13]. Neoplasms such as Burkitt lymphoma and gastric carcinoma only express EBV nuclear antigen 1 (EBNA1) and several non-coding RNAs (BamHI A rightward transcripts (BARTs) and EBV-encoded small RNAs (EBERs)) (latency I), whereas some Hodgkin lymphomas, nasopharyngeal carcinomas, and T/NK lymphomas, express EBERs, BARTs, EBNA1, latent membrane protein 1 (LMP1) and LMP2 genes (latency II). In addition to the latency II genes, EBNA2, EBNA3 and EBNA-LP are also expressed in immunosuppression-related lymphomas and lymphoblastoid cell lines (latency III) [13]. The transforming and oncogenic potential of the viral oncogenic protein LMP1 has been characterized both *in vivo* and *in vitro*. In a mouse model, LMP1 cooperated with LMP2A to induce the development of invasive carcinomas [14]. The immortalization of B-cells by EBV is also dependent on LMP1, which mimics the function of a constitutively active CD40 receptor [15] and activates NFκB, AKT, MAPK and c-Jun N-terminal kinase (JNK) signaling pathways [16]. Interestingly, LMP1 has been detected in exosomes (small secreted vesicles containing proteins, mRNAs and microRNAs), which can modulate the microenvironment [17]. Although LMP1 plays a pivotal role in the viral tumorigenesis of several EBV-mediated malignancies, LMP1 has not been detected in EBV-positive gastric tumors. It is not clearly understood why LMP1-positive cells are not detected in EBV-associated gastric cancer or how LMP1 affects the malignant transformation of gastric epithelial cells.

In this study, we found that the presence of neighboring LMP1-negative cells attenuated the growth of LMP1-positive cells. Furthermore, LMP1-positive cells enhanced the proliferation of surrounding LMP1-negative cells via exosomes. We demonstrated that LMP1-positive cells were apically extruded from a monolayer of gastric cancer-derived AGS cells, indicating cell competition between LMP1-positive and -negative cells.

## RESULTS

### LMP1 expression decreases gradually in AGS cells infected with EBV

**Figure 1:**
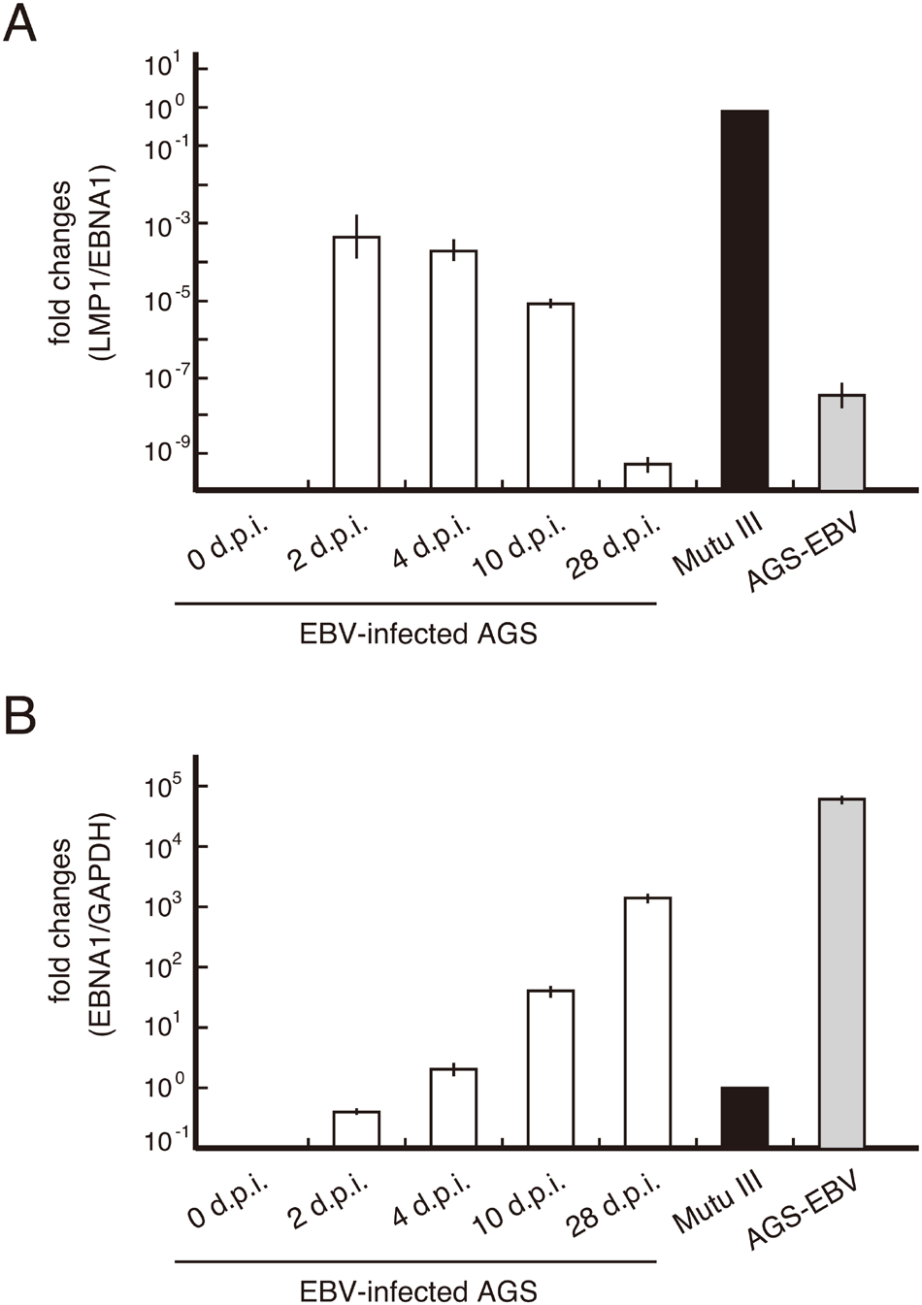
LMP1 mRNA expression decreased gradually in AGS cells infected with EBV AGS-CR2 cells were infected with EBV prepared from AGS-EBV cells. Total RNA was collected 0, 2, 4, 10 and 28 days post-infection (d.p.i.) and subjected to qRT-PCR. Relative mRNA levels of LMP1 **(A)** and EBNA1 **(B)** in Mutu III cells expressing the full latency III pattern of viral gene expression were determined after normalization to EBNA1 levels (for infected cells) and GAPDH level (for total cells).

A previous study found that a subpopulation of EBV-infected gastric cancer cells showed a latency II pattern of viral gene expression (including EBNA1, LMP1, LMP2 and EBERs) 3 days post-infection [18]. To monitor the expression of LMP1 in EBV-infected AGS gastric cancer cells, AGS cells stably expressing viral receptor CR2 (AGS-CR2) [19] were infected with enhanced green fluorescent protein (EGFP)-EBV [20] and maintained in selection medium containing G418 for isolation of infected cells. Quantitative RT-PCR (qRT-PCR) revealed that LMP1 mRNA was expressed during the early phase of EBV infection (Figure 1A) when the infected cells were surrounded by uninfected cells. LMP1 expression decreased gradually during selection as the number of infected cells increased (Figure 1A and 1B). Consistent with this result, evidence to date suggests that EBV-infected AGS cells express very low levels of LMP1 [21, 22]. These findings suggest that two populations of AGS cells exhibiting the latency I or II program are present during primary infection of AGS cells. Consequently, among EBV-infected cells, those exhibiting the latency I program (LMP1-negative cells) become dominant over LMP1-positive cells. Thus, it is reasonable to assume that the interaction between these two populations occurred within a heterogeneous cell community.

### Co-culture of LMP1-expressing and -nonexpressing cells suppresses LMP1-mediated growth

**Figure 2:**
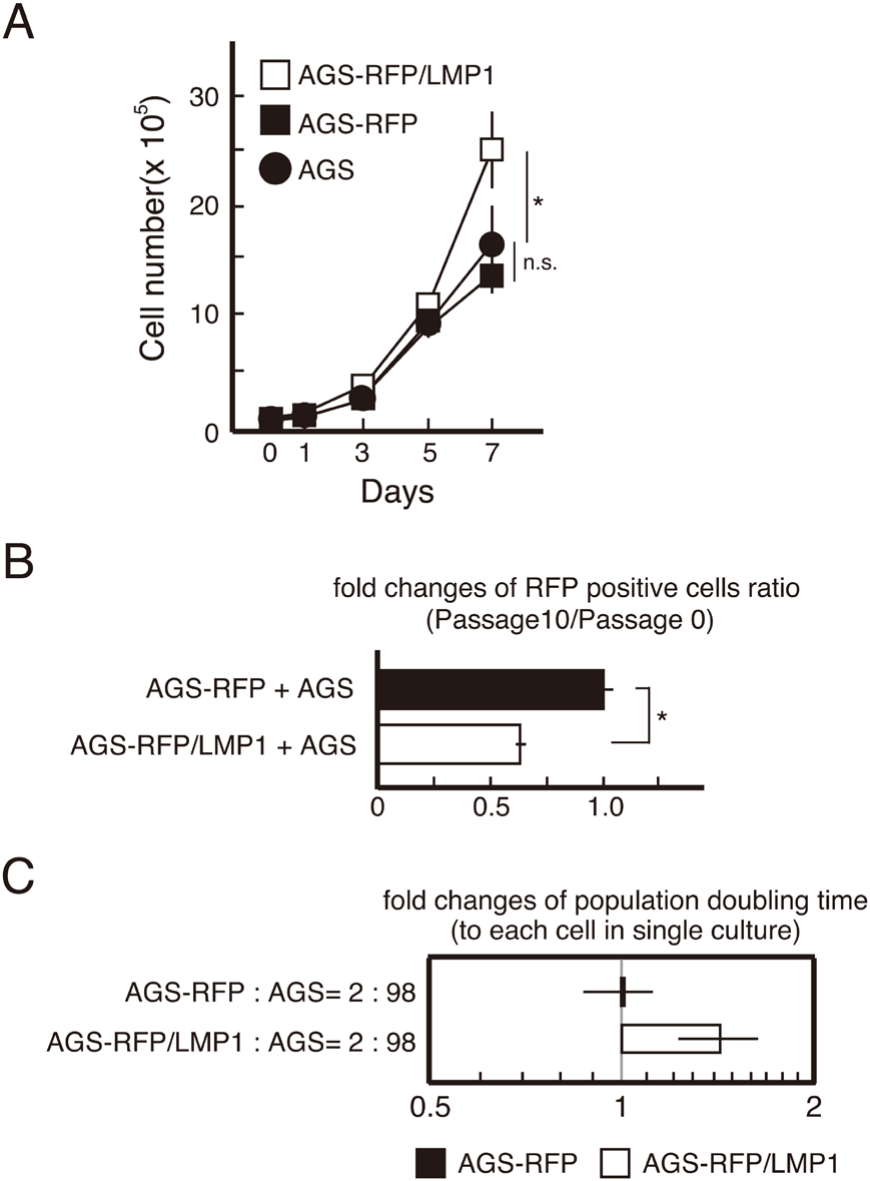
The number of LMP1-positive AGS cells decreased with each passage when co-cultured with LMP1-negative AGS cells **(A)** Enhancement of cell proliferation by LMP1 occurs in a cell-autonomous manner. Growth curve assays for AGS-RFP/LMP1, AGS-RFP and AGS cells are presented. * *P* < 0.05; n.s.: not significant (*P* > 0.05). **(B)** The LMP1-induced increase in proliferation was suppressed when LMP1-positive cells were surrounded by LMP1-negative cells. AGS-RFP/LMP1 or AGS-RFP cells were mixed with AGS cells at a ratio of 2:98 and cultured over 10 passages. The number of RFP-positive cells was compared between passages 0 and 10. Values are expressed as ratios relative to AGS-RFP+AGS cell numbers. * *P* < 0.05. **(C)** The population doubling time of LMP1-positive cells increased upon co-culturing with LMP1-negative cells. The population doubling times of AGS-RFP and AGS-RFP/LMP1 cells in monocultures and AGS cell co-cultures were determined. Values are expressed as ratios relative to the population doubling time in monocultures.

To investigate events at the boundary of LMP1-expressing and -nonexpressing cells, AGS cells that were labeled by red fluorescent protein (RFP) were transduced with LMP1 to establish LMP1-expressing cells (AGS-RFP/LMP1 cells). LMP1 expression enhanced cell proliferation in a cell-autonomous manner (Figure 2A), consistent with earlier reports [23]. To examine if the LMP1-mediated growth advantage was maintained when LMP1-positive cells were co-cultured with LMP1-negative cells, we mixed AGS-RFP/LMP1 or AGS-RFP cells (expressing RFP only) with AGS cells (do not express LMP1) at a ratio of 2:98. The two cell lines were co-cultured over 10 passages, and the number of RFP-positive cells was counted. Compared with AGS-RFP (LMP1-negative) cells, the number of AGS-RFP/LMP1 (LMP1-positive) cells decreased by passage 10 (Figure 2B), indicating LMP1-mediated cell growth was suppressed in a non-cell-autonomous manner, and/or the growth of surrounding LMP1-negative cells increased. To further assess the growth properties of the cells, the population doubling time for each cell line was calculated. While there was no difference in the population doubling time between AGS-RFP cells co-cultured with AGS cells and monocultures, the population doubling time of AGS-RFP/LMP1 cells co-cultured with AGS cells was 1.5-fold greater than that of monocultures (Figure 2C). These findings suggest that the presence of surrounding LMP1-negative cells reduced the number of LMP1-positive cells.

### LMP1-expressing cells are eliminated from a monolayer of AGS cells

**Figure 3:**
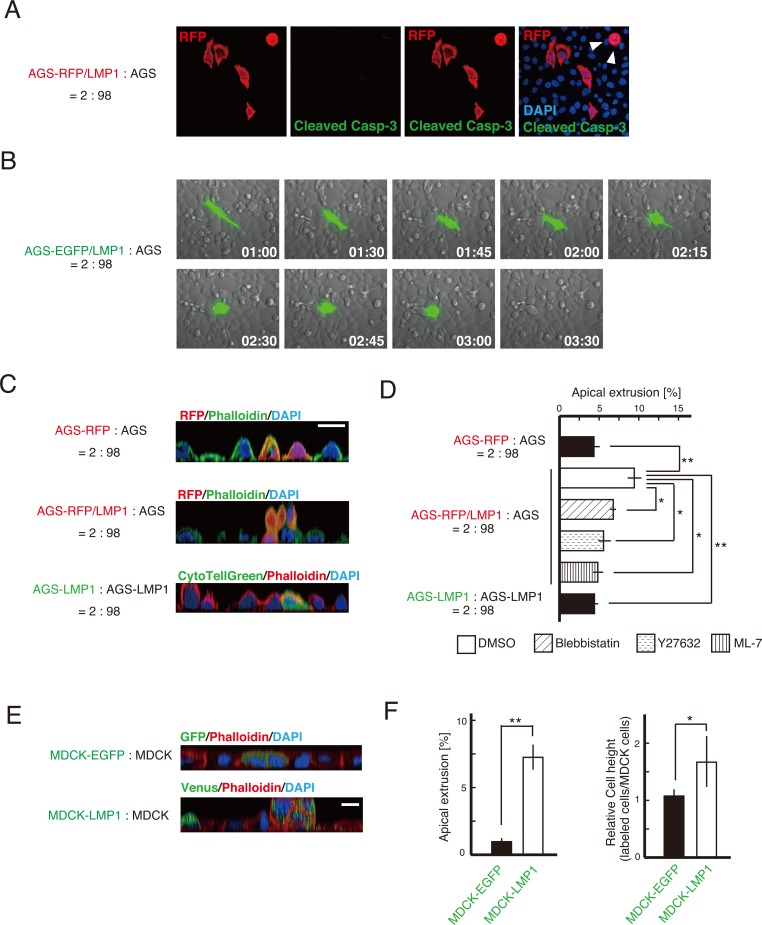
LMP1-positive cells were eliminated from an AGS cell monolayer **(A)** Caspase-activated apoptotic cells were not detected when LMP1-expressing AGS cells were co-cultured with LMP1-negative AGS cells. AGS-RFP/LMP1 cells were cultured with AGS cells at a ratio of 2:98. Caspase-activated apoptotic cells were visualized by anti-cleaved caspase-3 antibody. Cleaved caspase 3-positive cells were not detected in either rounded or non-rounded cells. Arrowheads indicate LMP1-positive cells with a round shape. **(B)** LMP1-positive cells were apically extruded when surrounded by LMP1-negative cells. AGS-EGFP/LMP1 or AGS-EGFP cells were cultured with AGS cells at a ratio of 2:98 on a glass-bottom dish. Images are representative time-lapse images of AGS-EGFP/LMP1 cells. **(C** and **D)** Confocal microscopic z-sections of AGS-RFP/LMP1 cells surrounded by AGS cells (**C**; middle panel) or AGS-RFP/LMP1 cells (**C**; lower panel). The RFP-labeled cells (or transiently fluorescently labeled cells) were mixed and cultured as indicated, followed by staining with phalloidin and DAPI. Scale bar, 20 μm. The number of labeled cells extruded apically from AGS cell monolayers in the presence of inhibitors was counted **(D)**. Data are presented as means ± standard error from three independent experiments. For each experiment, 70-300 cells were counted. ** *P* < 0.01; * *P* < 0.05. **(E)** The fate of LMP1-positive MDCK cells surrounded by normal MDCK cells. MDCK cells were transiently transfected with the LMP1-IRES-Venus expression plasmid or control EGFP expression plasmid. Cells were fixed and stained with an anti-GFP antibody (green), phalloidin (red) and DAPI (blue). Scale bar, 10 μm. **(F)** Quantification of cell height and apical extrusion of LMP1-positive MDCK cells. Data are presented as means ± standard error from of independent experiments. ** *P* < 0.01; * *P* < 0.05.

To understand why the population of LMP1-positive cells decreased upon co-culturing with LMP1-negative cells, we first investigated whether LMP1-expressing cells underwent apoptosis within the AGS cell monolayer. AGS-RFP/LMP1 cells were mixed with AGS cells at a ratio of 2:98, fixed and incubated with an antibody detecting cleaved caspase-3, a marker of cell death. Detection of activated caspase-3 showed that the LMP1-positive cells adjacent to LMP1-negative cells did not undergo cell death (Figure 3A). A similar result was obtained in cells stained for cleaved PARP, another apoptotic marker (data not shown). Of note, a few AGS-RFP/LMP1 cells surrounded by AGS cells exhibited a rounded morphology (arrowheads in Figure 3A). This finding indicates that the decrease in the population of LMP1-positive cells surrounded by LMP1-negative cells was possibly caused by the elimination of LMP1-positive cells from the mixed cell population. A similar pattern of abnormal cell elimination from the epithelium was reported during competition between Ras^V12^- or Src-transformed and normal MDCK cells [2, 24]. To analyze the dynamics of cell elimination directly, we observed the fate of LMP1-positive cells surrounded by LMP1-negative cells using time-lapse microscopy. LMP1-expressing cells were extruded from the apical surface of a monolayer of LMP1-nonexpressing cells (Figure 3B and Supplementary Movie 1), although this apical extrusion was not observed in control AGS-RFP cells (Figure 3C and 3D). As shown in the confocal microscopic z-sections in Figure 3C, the LMP1-positive cells were indeed delaminated apically. Moreover, fluorescently labeled LMP1-positive cells were not extruded when mixed with non-labeled LMP1-positive cells (Figure 3C and 3D), indicating that the extrusion of LMP1-positive cells depends on the presence of surrounding LMP1-negative cells. To investigate the mechanism involved in this phenomenon, we examined the effect of inhibitors of the Rho/myosin-II pathway, since this pathway plays a vital role in apical extrusion of transformed cells [2, 3]. Blebbistatin, Y27632 and ML-7, which inhibit myosin-II, ROCK and MLCK, respectively, moderately suppressed apical extrusion of LMP1-positive cells co-cultured with LMP1-negative cells (Figure 3D and Supplementary Figure 1). These results suggest that the Rho/myosin-II pathway is at least partially involved in this process.

Furthermore, we examined whether apical extrusion of LMP1-positive cells occurs in other cell lines. MDCK cells were transiently transfected with the pcDNA4-LMP1-IRES-Venus plasmid, which enabled to label the LMP1-expressing cells by fluorescent protein Venus, and mixed with normal MDCK cells. The LMP1-expressing cells were apically extruded, and the height of the LMP1-positive cells was increased compared with that of the LMP1-negative neighboring cells (Figure 3E and 3F). These findings suggest that LMP1-positive cells compete with LMP1-negative cells, resulting in their elimination from co-cultures.

### LMP1-expressing cells stimulate the proliferation of surrounding LMP1-negative cells via exosomes

**Figure 4:**
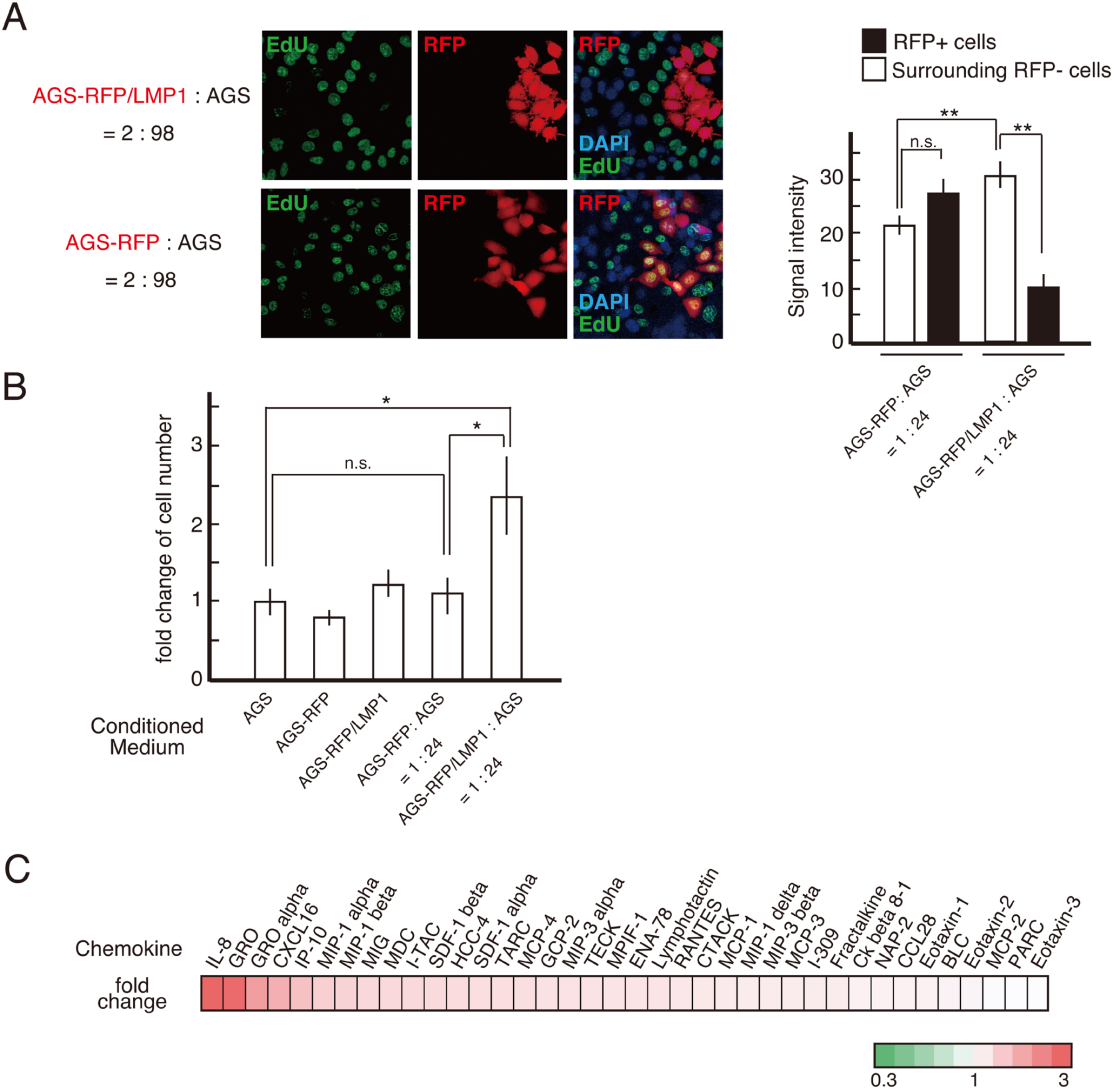
LMP1-positive cells stimulated the proliferation of surrounding AGS cells **(A)** LMP1-positive cells enhanced the proliferation of surrounding AGS cells. AGS-RFP/LMP1 cells were mixed with AGS cells at a ratio of 2:98 and then seeded on coverslips. Cells were labeled with EdU, and EdU incorporation (green) was determined in combination with anti-RFP antibody staining (red). Nuclei were stained with DAPI (blue). The signal intensity of EdU in surrounding RFP-negative cells was compared to that of RFP-positive cells. ** *P* < 0.01; n.s.: not significant (*P* > 0.05). **(B)** CM from the AGS-RFP/LMP1 cells co-cultured with AGS cells enhanced the proliferation of AGS cells. Recipient AGS cells were maintained in RPMI-1640 medium supplemented with 0.1% FBS for 24 h and then treated with CM derived from the AGS-RFP/LMP1 or AGS-RFP cells co-cultured with AGS cells, AGS-RFP/LMP1 cells, AGS-RFP cells, or AGS cells. After 72 h of incubation, cells were harvested and counted. Values are expressed as the fold change in cell number relative to that in the control treatment (fresh medium). * *P* < 0.05; n.s.: not significant (*P* > 0.05). **(C)** Chemokine profiles in CM. Chemokine levels in CM were monitored by hybridization of CM to a human chemokine antibody array. Chemokines are ranked according to the magnitude of the fold change expression in CM from AGS-RFP/LMP1+AGS cells relative to CM from AGS-RFP+AGS cells. The ratios were calculated using the mean chemiluminescence intensities of the corresponding protein spots after background correction and normalization to the mean intensities of the positive controls. Values greater than 1 are displayed in red; values less than 1 are displayed in green. The numbers on the heat map key (bottom) indicate log2-fold changes between the two groups.

**Figure 5:**
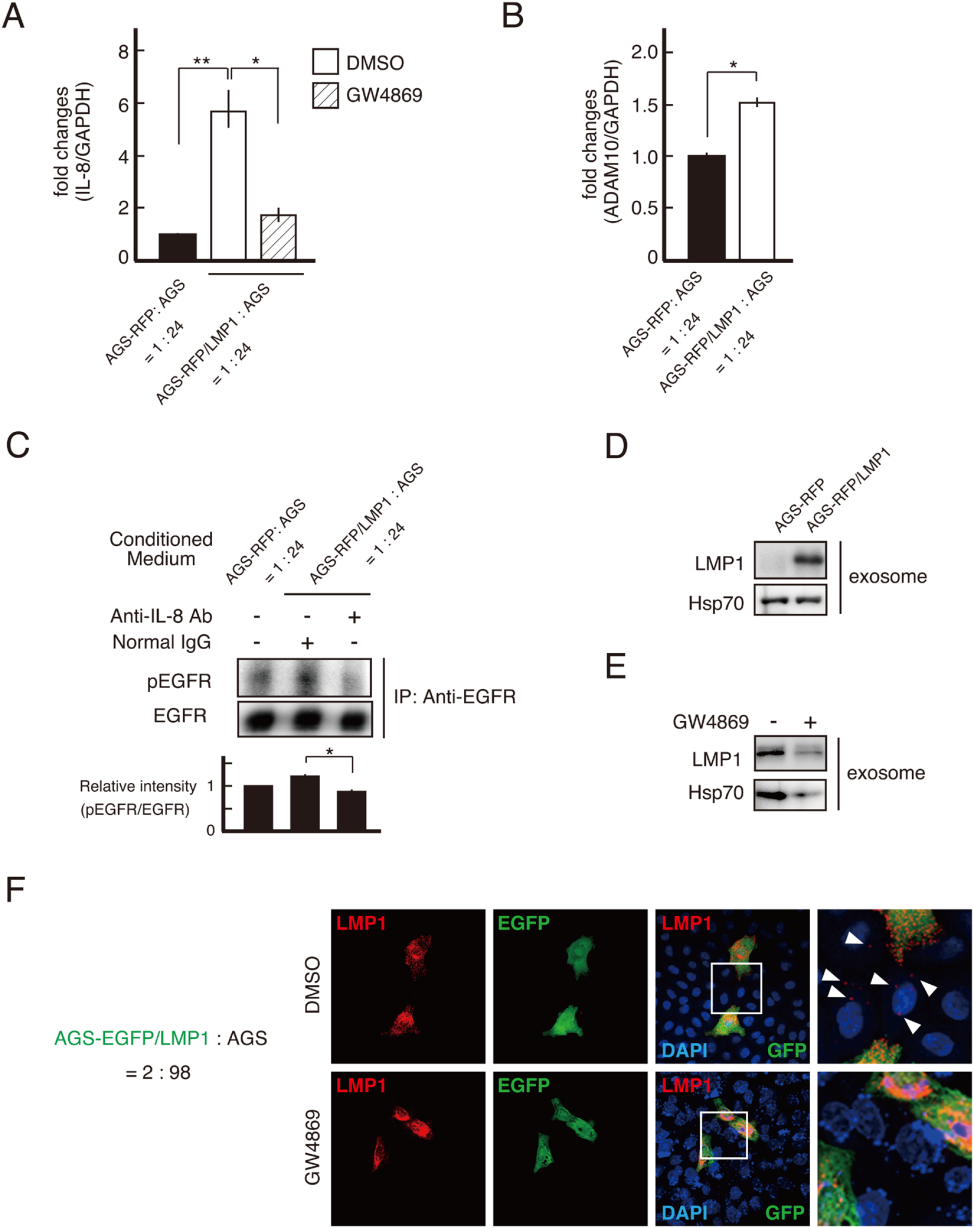
LMP1-containing exosomes upregulated IL-8 expression, driving EGFR phosphorylation in the surrounding cells **(A)** IL-8 expression was upregulated in AGS-RFP/LMP1 and AGS cell co-cultures. AGS-RFP/LMP1 or AGS-RFP cells were mixed with AGS cells at a ratio of 1:24 and cultured for 48 h in the presence of GW4869 (10 μM) or DMSO. RNA was extracted and subjected to qRT-PCR. Values are expressed as the fold change relative to that of the control after normalization to the housekeeping gene GAPDH. Data are means ± standard error from three to six independent experiments. ** *P* < 0.01; * *P* < 0.05. **(B)** ADAM10 expression was upregulated in AGS-RFP/LMP1 and AGS cell co-cultures. Samples were prepared as described in Figure 5A. Data are presented as means ± standard error from three independent experiments. * *P* < 0.05. **(C)** The effect of IL-8 on EGFR phosphorylation in AGS cells was induced by co-culturing with AGS-RFP/LMP1 cells. Cells were preincubated for 30 min with 8 μg/ml anti-IL-8 (to neutralize IL-8) or control antibody and then treated with CM to induce EGFR phosphorylation. The band intensities of phosphorylated EGFR were quantified and normalized to those of EGFR. * *P* < 0.05. (**D** and **E**) LMP1-positive cells produced exosomes containing LMP1. Exosomes were isolated from the supernatant of LMP1-positive or -negative cells **(D)** and from the supernatant of AGS-RFP/LMP1 cells in the presence of GW4869 (10 μM) or DMSO **(E)**. Hsp70 was used as an exosome marker. **(F)** LMP1-containing exosomes secreted from LMP1-positive cells spread to surrounding LMP1-negative cells. AGS-EGFP/LMP1 cells were mixed with AGS cells at a ratio of 2:98 and then cultured on coverslips in the presence of GW4869 (10 μM) or DMSO. Cells were fixed and stained for LMP1 and EGFP using specific antibodies. Nuclei were stained with DAPI. Arrowheads indicate transported LMP1 in surrounding AGS cells.

Although it remains to be elucidated which cells outcompete others in cell competition [25], a difference in the proliferation rate is a trigger in cell competition [26]. We next examined the cell growth of each co-cultured cell line using ethynyl deoxyuridine (EdU) incorporation. Elevated DNA synthesis was observed in LMP1-negative cells surrounding LMP1-positive cells (Figure 4A). This result was also confirmed by staining for the proliferation marker Ki-67 (data not shown). Thus, at the boundary of LMP1-positive and -negative cells, the proliferation of LMP1-negative cells was stimulated. This suggests cell growth was stimulated by paracrine signaling at the interface between LMP1-positive and -negative cells. To investigate this possibility, AGS cells were exposed to conditioned medium (CM) from the LMP1-positive cells co-cultured with LMP1-negative cells. As shown in Figure 4B, CM collected from co-cultures comprising LMP1-positive (AGS-RFP/LMP1) and -negative (AGS) cells stimulated the proliferation of recipient AGS cells. On the other hand, treatment with control CM from AGS-RFP cells, AGS-RFP/LMP1 cells alone or AGS cell co-cultures (AGS-RFP+AGS) did not significantly increase cell proliferation compared with treatment with medium (Figure 4B). We also confirmed that CM from AGS-RFP/LMP1 + AGS cells enhance the growth of AGS cells in a dose-dependent manner (Supplementary Figure 2). These findings supported the possibility that a secreted soluble factor stimulated the proliferation of surrounding LMP1-negative cells in the co-culture system. Furthermore, to identify paracrine effectors secreted upon interaction of AGS-RFP/LMP1 with AGS cells, we used the Human Chemokine Antibody Array for 38 chemokines and found that IL-8 expression was upregulated (Figure 4C and 5A). Since previous studies showed that IL-8-stimulated AGS proliferation was mediated by epidermal growth factor receptor (EGFR) transactivation via a disintegrin and metalloproteinase (ADAM) 10 in *Helicobactor pylori* infection [27, 28], we next examined the levels of ADAM10 expression and EGFR phosphorylation. As shown in Figure 5B, the expression of ADAM10 was upregulated in LMP1-positive and -negative cell co-cultures. Moreover, blocking IL-8 with a neutralizing antibody suppressed EGFR phosphorylation, which was stimulated by CM from AGS-RFP/LMP1 cells co-cultured with AGS cells (Figure 5C). We confirmed that CM collected from co-cultures enhanced EGFR phosphorylation in a dose-dependent manner (Supplementary Figure 3). These results suggest that IL-8 enhances LMP1-negative cell proliferation via EGFR transactivation. LMP1-expressing cells release LMP1-containing exosomes, which are taken up by neighboring cells, leading to the modulation of various signaling pathways in the recipient cells [29, 30]. Intriguingly, treatment with GW4869, an inhibitor of sphingomyelinase that markedly reduces exosome secretion [31], attenuated IL-8 expression (Figure 5A). This finding suggests that stimulation of IL-8 expression occurred in a non-cell-autonomous manner. Consistent with these findings, we found that exosomes secreted from LMP1-positive cells contained LMP1 protein (Figure 5D), and GW4869 inhibited the secretion of LMP1-containing exosomes (Figure 5E). As shown in Figure 5F, LMP1-negative (EGFP-negative) cells surrounding LMP1-positive (EGFP-positive) cells exhibited punctate LMP1 signals in the cytoplasm, and such punctate LMP1 signals were not observed when the cells were treated with GW4869. These observations strongly support the idea that LMP1 protein is exosomally transferred from LMP1-positive cells to LMP1-negative cells. However, we cannot rule out the possibility that a molecule other than LMP1 in exosomes triggered IL-8 expression. Taken together, our findings suggest that LMP1-containing exosomes secreted from LMP1-positive cells spread to surrounding LMP1-negative cells to induce IL-8 expression in recipient cells, leading to enhanced proliferation of LMP1-negative cells through EGFR transactivation.

## DISCUSSION

In this study, we showed that during the early phase of EBV infection of gastric cancer-derived AGS cells, a subpopulation of LMP1-expressing cells disappeared among EBV-infected cells. EBV-infected AGS cells expressed the latency type I program, but not the type II program. Similar to AGS cells, LMP1 expression was detected during the early phase of primary infection in other gastric tissue-derived cell lines, but expression was almost undetectable upon isolation of cells latently infected with EBV (personal communication with Dr. Nishikawa (Yamaguchi University, Japan)). The robustness of the observed correlation between LMP1 expression and isolation of several EBV-infected gastric cell lines supports a potential functional relationship between two cell populations expressing latency I or II programs. When these two populations of cells are co-cultured, the latency I program cells (LMP1-negative cells) become dominant over the LMP1-positive cells. Moreover, we demonstrated that LMP1-positive AGS cells were eliminated from a monolayer of AGS cells when surrounded by LMP1-negative cells and this apical extrusion of LMP1-positive cells was inhibited in the presence of surrounding LMP1-positive cells (Figure 3B, 3C and 3D), suggesting cell competition between LMP1-positive and -negative cells. Thus, our findings indicate cell competition as a mechanism driving the suppression of viral oncogene expression within a heterogeneous cell population. Most previous studies have focused on intrinsic factors such as transcription factors that regulate the viral latency program [revied in [13]]. In line with these studies, we find it interesting that extrinsic factors such as microenvironmental conditions and neighboring cells can also influence viral latency.

The role of LMP1 in the transformation of gastric epithelia remains unclear. Recently, the genetics of EBV-negative compared with EBV-positive gastric cancer were characterized by the Cancer Genome Atlas Research Network. The authors identified distinct mutations and epigenetic profiles (recurrent PIK3CA mutations, high DNA hypermethylation levels and amplification of JAK2, CD274 and PDCD1LG2) in EBV-associated gastric cancer cases [11]. Since LMP1 induces promoter hypermethylation via activation of DNA methyltransferase 1 [32] and the polycomb group protein Bmi-1 [33], LMP1 may contribute to global methylation and epigenetic silencing of multiple cancer genes during the initial stages of EBV-associated gastric cancer. On the other hand, Raab-Traub's group reported that inhibition of LMP1 expression did not affect growth or alter gene expression profiles in an established gastric cancer cell line [21], suggesting that LMP1 is largely dispensable in transformed gastric epithelial cells. Therefore, LMP1-positive gastric cells show lower fitness within tissues than do other EBV-associated cancer cells and thus are eliminated from the gastric epithelium when surrounded by normal cells. These findings correlate with the expression pattern of LMP1 in primary EBV infection of gastric epithelial cells, as shown in Figure 1.

The cells eliminated by cell competition are often slower growing than their competitors. Our findings indicate that a difference in the proliferation rate led to cell competition between LMP1-positive and -negative cells when these cells were co-cultured. Recent work evaluating HSV-1 with an extremely low multiplicity of infection showed that the surrounding uninfected cells had elevated rates of host cell DNA synthesis, which was mediated by paracrine mediator(s) secreted from HSV-infected cells [34]. Thus, at an early stage of viral infection, in addition to innate and adaptive immunity, uninfected cells possibly provide intrinsic immunity against infected cells. In this study, we demonstrated that exosomes secreted from LMP1-positive cells induced IL-8 expression, leading to phosphorylation of EGFR (Figure 5A and 5C), consistent with the enhanced EdU uptake detected in surrounding LMP1-negative cells (Figure 4A). GW4869, which inhibits exosome secretion, attenuated IL-8 upregulation (Figure 5A), suggesting that elevated IL-8 expression occurred in the recipient cells rather than in the LMP1-positive cells. We cannot rule out the possibility that additional factors present in the exosomes secreted from the LMP1-positive cells affected IL-8 upregulation. Nevertheless, these results indicate that paracrine factors stimulate proliferation of the LMP1-negative cells surrounding LMP1-positive cells. However, it is unclear why paracrine effects on cell proliferation are suppressed in the LMP1-positive cells or how the LMP1-positive cells are apically extruded from AGS cell monolayers. To clarify these points, further studies are required.

In summary, we showed that cell-cell communication led to elimination of cells expressing the viral latent gene LMP1 from a heterogeneous cell population consisting of LMP1-negative cells, suggesting that viral convergent latency is established in a cell-autonomous and non-cell-autonomous manner.

## MATERIALS AND METHODS

### Plasmids, cells and reagents

Full-length cDNAs for LMP1 (EBV Akata strain), RFP and EGFP were obtained by RT-PCR. LMP1 was subcloned into the pcDNA3.1(+) vector (Invitrogen), and RFP and EGFP were subcloned into the pcDNA4/TO/myc-His vector (Invitrogen). A LMP1 fragment and IRES2-Venus fragment produced from the CSII–CMV–MCS–IRES2–Venus plasmid, which was kindly provided by Dr. Miyoshi (RIKEN BioResource Center), were inserted into the pcDNA4/TO/myc-His vector (Invitrogen). The inserted DNA sequence of each vector was confirmed by DNA sequencing.

The AGS (ATCC: CRL-1739), AGS-CR2 [19], AGS-EBV [19, 20] and Mutu III [35] cell lines were maintained in RPMI-1640 medium supplemented with 10% fetal bovine serum (FBS). MDCK (NBL-2) cells (JCRB: JCRB9029) were maintained in DMEM supplemented with 10% FBS. AGS-RFP and AGS-EGFP cells were established by transfecting linearized pcDNA4-RFP and pcDNA4-EGFP, respectively, into AGS cells and selecting for transfected cells using zeocin. AGS-RFP/LMP1 and AGS-EGFP/LMP1 cells were established by transfecting linearized pcDNA3-LMP1 into AGS-RFP and AGS-EGFP cells, respectively, and selecting for transfected cells using G418. For EBV infection of AGS cells, AGS-CR2 cells were infected with EGFP-EBV [20] prepared from the culture supernatant of AGS-EBV cells. EGFP-positive cells comprised approximately 0.5% of AGS-CR2 cells at 2 days post-infection (d.p.i.). G418 was added to medium from 3 d.p.i. to select infected cells.

Anti-GFP (598) rabbit polyclonal antibody and anti-RFP (8D6) mouse monoclonal antibody were purchased from MBL. Anti-cleaved caspase-3 (5A1E) rabbit monoclonal antibody, rabbit anti-EGFR (D38B1), rabbit anti-phospho-EGFR (D7A5) and horseradish peroxidase-conjugated secondary antibodies were obtained from Cell Signaling Technology. Rabbit anti-EGFR (EP38Y: Abcam), mouse anti-CXCL8/IL-8 (6217: R&D Systems), mouse anti-LMP1 (CS1-4: DAKO), mouse anti-Hsp70 (C92F3A-5: Enzo Life Sciences) and normal mouse IgG (sc-2025: Santa Cruz Biotechnology) antibodies were also used. Secondary goat anti-mouse or rabbit IgG antibodies conjugated with Alexa Fluor 488 or 546 and Alexa Fluor 647-conjugated phalloidin were purchased from Thermo Fisher Scientific.

For inhibition of exosome secretion, cells were treated with 10 μM GW4869 (Cayman) for 24–48 h before harvesting. (S)-(−)-blebbistatin (30 μM; Toronto Research Chemicals), Y27632 (10 μM; Cayman) and ML-7 (10 μM; Cayman) were used to inhibit the Rho/myosin-II pathway. For fluorescent labeling of the cells, CytoTell UltraGreen (AAT Bioquest) was used according to the manufacturer's instructions.

### Growth curves and population doubling time

Cells were seeded at a density of 1×10^4^/well in a 12-well plate. Every 48 h, cells were harvested by trypsinization, and the number of viable cells was counted using the trypan blue exclusion method. To determine the population doubling times, RFP-positive cells were mixed with AGS cells at a ratio of 2:98. The mixed cells were seeded at a density of 1×10^5^/60-mm culture dish. Cells were harvested every 24 h for 3–5 days. The number of viable cells was counted using the trypan blue exclusion method, and the number of RFP-positive cells was estimated by FACS analysis. The population doubling time was calculated from the cell growth curve. The equation used to calculate the doubling time is as follows: (t-t_i_)log2/logN-logN_i_ (t, final time; t_i_, initial time; N, final cell number; N_i_, initial cell number).

### Co-culture assay

For population analysis, RFP-positive cells were mixed with AGS cells at a ratio of 2:98. The mixed cells were maintained in RPMI-1640 medium supplemented with 10% FBS. At passages 0 and 10, the percentage of RFP-positive cells was determined by microscopy.

For the CM assays, 1×10^6^ mixed cells were seeded in a 10-cm dish on day 0. The cell medium was replaced with fresh medium on days 1 and 3, and this medium was considered CM. AGS cells were incubated with RPMI-1640 medium supplemented with 0.1% FBS for 24 h. AGS cell were seeded at a density of 1×10^4^/well in a 24-well plate and incubated with 800 μl RPMI-1640 medium supplemented with 0.2% FBS plus 200 μl filtered CM derived from either AGS-RFP + AGS cells or AGS-RFP/LMP1 + AGS cells. After 72 h of incubation, cells were harvested, and the number of viable cells was counted as described above.

### Immunofluorescence staining

RFP- or EGFP-expressing cells were mixed with AGS cells at a ratio of 2:98 and seeded on coverslips. After 12 h to 3 days, cells were washed with PBS and fixed with either 4% paraformaldehyde for 15 min at room temperature followed by permeabilization with 0.1% Triton X-100 or ice-cold ethanol/acetone for 20 min at −20°C. Samples were blocked using BlockAid Blocking Solution (Thermo Fisher Scientific) for 2 h at room temperature. The cells were incubated with primary anti-cleaved caspase-3 (1:100), anti-RFP (1:250), anti-GFP (1:250) and anti-LMP1 (1:50) antibodies. The secondary antibodies were applied at a 1:200 dilution. All washes following the antibody incubations were performed using TBS containing 0.01% Tween 20. For actin staining, Alexa Fluor 647-conjugated phalloidin was used according to the manufacturer's instructions. Coverslips were mounted using the ProLong Diamond antifade reagent with DAPI (Thermo Fisher Scientific). Images were captured and processed using a LSM880 confocal laser microscope (Zeiss) with ZEN microscope software (Zeiss). The EdU incorporation assay was performed using the Click-IT PLUS EdU Alexa Fluor 488 Imaging kit (Thermo Fisher Scientific) according to the manufacturer's protocol (EdU was incubated at a final concentration of 5 μM for 30 min).

### qRT-PCR

Total RNA was isolated from cells and converted into cDNA using the Superprep Cell Lysis & RT kit for qPCR (TOYOBO). Real-time PCR was performed on the Stratagene Mx3000p qPCR system (Agilent Technologies) using SYBR Premix ExTaq II (TaKaRa). The threshold cycle (Ct) value was normalized to GAPDH (for cellular genes) or EBNA1 (for viral genes), and the relative fold change was computed using the ΔΔCt method. The primer sequences used were 5′-CTGGCCGTGGCTCTCTTG-3′ and 5′-CCTTGGCAAAACTGCACCTT-3′for IL-8; and 5′-GGAAGATGGTGTTGCTGAGAG-3′ and 5′-ACGCTGGTGTTTTTGGTGTAA-3′ for ADAM10. The other primer sequences used were described previously [36].

### Time-lapse imaging

AGS-EGFP/LMP1 or AGS-EGFP cells were mixed with AGS cells at a ratio of 2:98 and seeded on 35-mm glass-bottom culture dishes (μ-Dish; Ibidi). Mixed cells were incubated for 12–24 h until a monolayer formed. For time-lapse imaging, cells were monitored using the LCV110 (Olympus) incubator fluorescence microscope. Images were captured every 5 min and analyzed using Metamorph digital analysis software (Universal Imaging).

### Membrane antibody array

The Human Chemokine Antibody Array-Membrane from Abcam (ab169812) was used to detect 38 chemokines simultaneously in cell culture supernatants. The staining was performing according to the manufacturer's instructions.

### EGFR tyrosine phosphorylation assay

Subconfluent AGS cells cultured in RPMI-1640 medium were switched to serum-free medium for 8 h and then treated with CM or fresh medium for 45 min. Anti-IL-8 antibody (to neutralize IL-8) was added to the cells for 30 min before stimulation. Cells were lysed with lysis buffer (137 mM NaCl, 2.7 mM KCl, 10 mM PO_4_^3−^, 1% Triton X-100 and 1 mM EDTA) containing protease and phosphatase inhibitor cocktails (Sigma) and then sonicated. The cell debris was removed by centrifugation, and the supernatant was used for immunoprecipitation assays using an anti-EGFR (EP38Y) antibody. Complexes of antibody and antigen were washed four times with lysis buffer. The immunoprecipitates were subjected to SDS-PAGE followed by immunoblot analysis using an anti-phospho-EGFR antibody.

### Purification of exosomes

Exosomes were purified using the ExoTrap Exosome Isolation Spin Column Kit (Cosmo Bio) according to the manufacturer's protocol.

### Statistical analysis

Values are expressed as means ± standard error from three independent experiments. Statistical analysis was performed using Microsoft Excel. Differences between the two groups were determined by Welch's *t*-test and were considered statistically significant at *P* < 0.05.

## SUPPLEMENTARY MATERIALS FIGURES AND TABLES




