# Supplementary material for: Elimination of LMP1-expressing cells from a monolayer of gastric cancer AGS cells
Source: Oncotarget. 2017 Apr 10;8(24):39345–55. doi: 10.18632/oncotarget.16996 (PMC5503617; doi:10.18632/oncotarget.16996)
Supplement: Supplementary file 1 [file oncotarget-08-39345-s001.pdf]

Elimination of LMP1-expressing cells from a monolayer of gastric cancer AGS cells

SUPPLEMENTARY INFORMATION

SUPPLEMENTARY FIGURES AND MOVIE

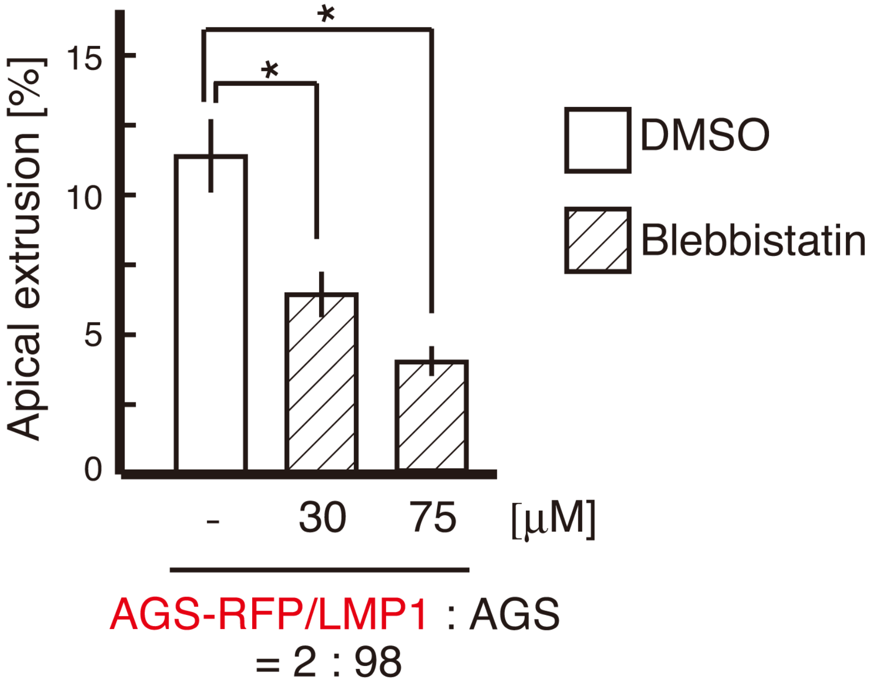

Supplementary Figure 1: Blebbistatin inhibited the apical extrusion of LMP1-positive cells in a dose-dependent manner. AGS-RFP/LMP1 cells were cultured with AGS cells at a ratio of 2:98. The number of RFP-positive cells extruded apically from AGS cell monolayers in the presence of blebbistatin (30 or 75 μM) was counted. Data are presented as means ± standard error from three independent experiments. For each experiment, 100-250 cells were counted. * $P < 0.05$.

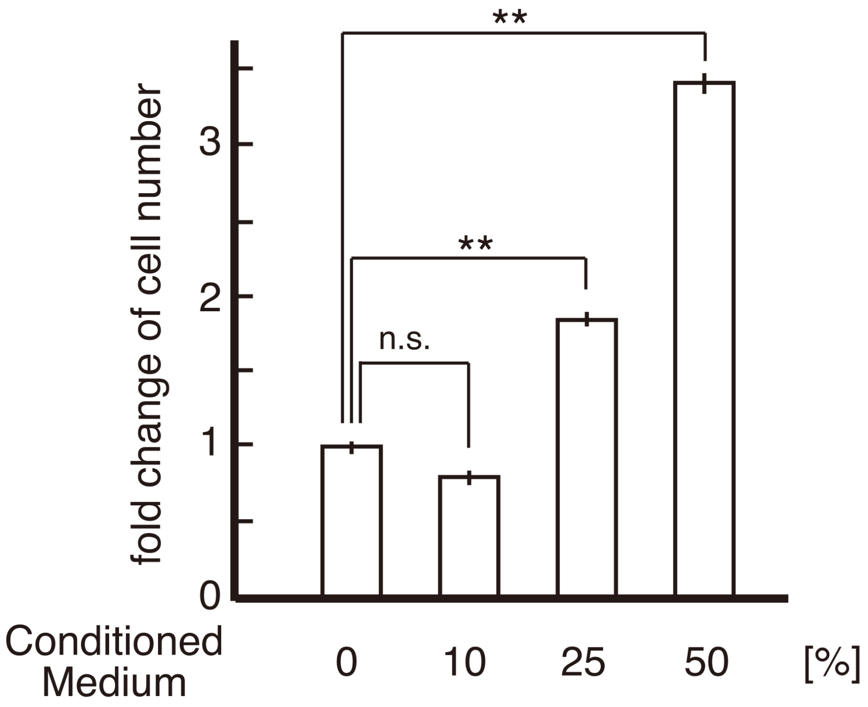

Supplementary Figure 2: CM increased the growth of AGS cells in a dose-dependent manner. Recipient AGS cells were maintained in RPMI-1640 medium supplemented with 0.1% FBS for 24 h and then treated with CM derived from the AGS-RFP/LMP1 cells co-cultured with AGS cells as indicated dose. After 72 h of incubation, cells were harvested and counted. Values are expressed as the fold change in cell number relative to that in the control treatment. ** $P < 0.01$; n.s.: not significant ($P > 0.05$).

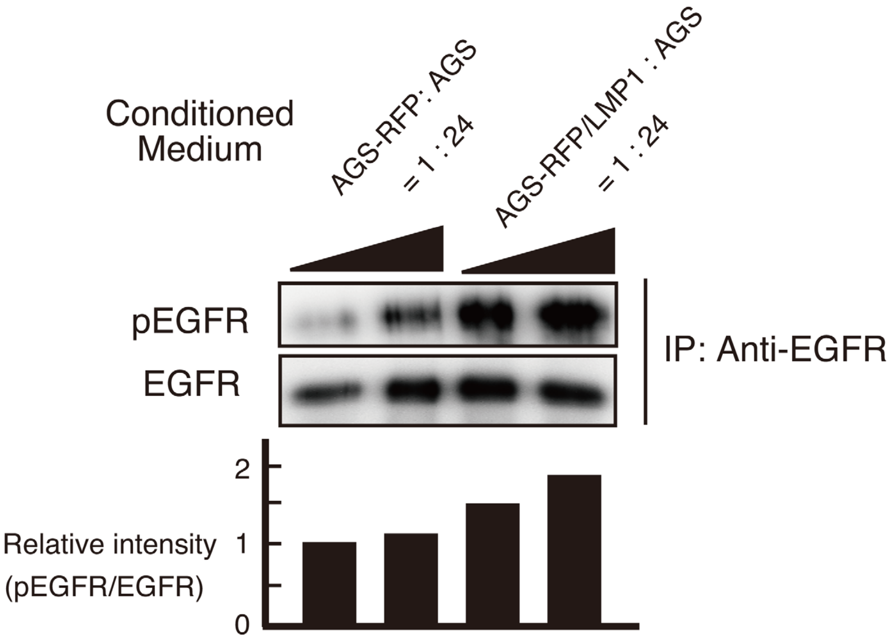

Supplementary Figure 3: CM increased the level of phosphorylated EGFR in a dose-dependent manner. Cells were treated with different doses of CM (150 and 500 μl) for 45 min and then harvested. Samples were probed with an antibody against phosphorylated EGFR following immunoprecipitation using an anti-EGFR antibody.

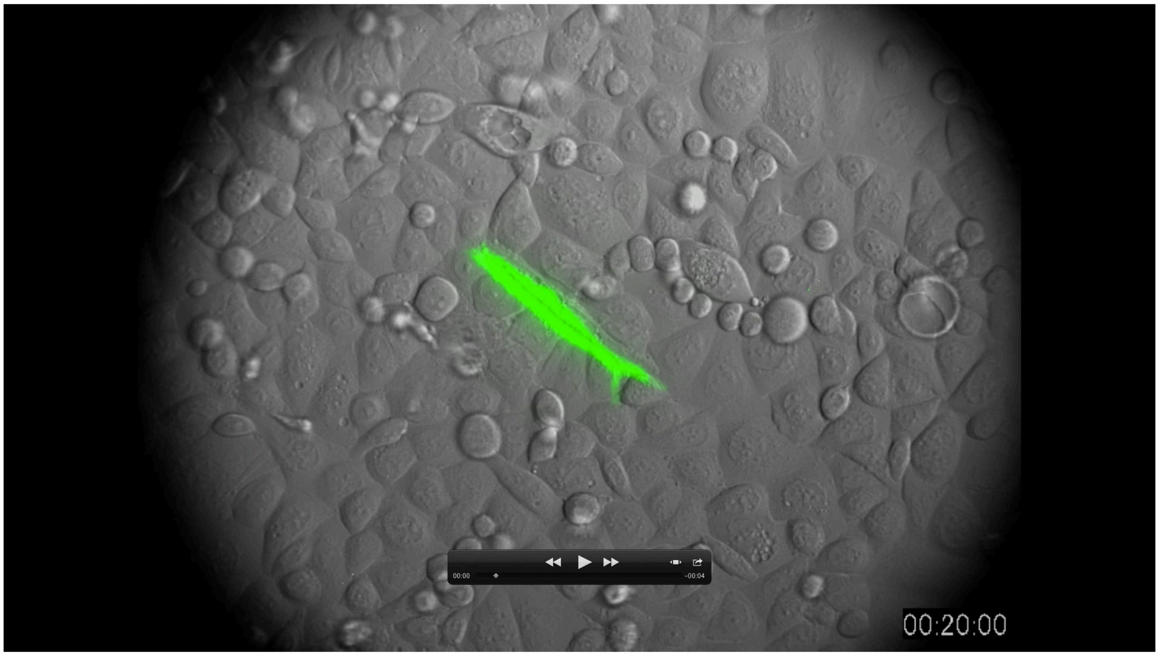

Supplementary Movie 1: The movie demonstrates the elimination of LMP1-positive cells from a monolayer of LMP1-negative AGS cells. AGS-EGFP/LMP1 cells were mixed with AGS cells at a ratio of 2:98 and then cultured on a glass-bottom dish. Once extruded, LMP1-positive cells migrated away above the AGS monolayer. Time-lapse images were captured at 5-min intervals.

See Supplementary Movie 1
